# Broadband Focusing Acoustic Lens Based on Fractal Metamaterials

**DOI:** 10.1038/srep35929

**Published:** 2016-10-26

**Authors:** Gang Yong Song, Bei Huang, Hui Yuan Dong, Qiang Cheng, Tie Jun Cui

**Affiliations:** 1 State Key Laboratory of Millimeter Waves, Department of Radio Engineering Southeast University, Nanjing 210096, People’s Republic of China; 2 College of Science, Nanjing University of Posts and Telecommunicaitons, Nanjing 210023, People’s Republic of China

## Abstract

Acoustic metamaterials are artificial structures which can manipulate sound waves through their unconventional effective properties. Different from the locally resonant elements proposed in earlier studies, we propose an alternate route to realize acoustic metamaterials with both low loss and large refractive indices. We describe a new kind of acoustic metamaterial element with the fractal geometry. Due to the self-similar properties of the proposed structure, broadband acoustic responses may arise within a broad frequency range, making it a good candidate for a number of applications, such as super-resolution imaging and acoustic tunneling. A flat acoustic lens is designed and experimentally verified using this approach, showing excellent focusing abilities from 2 kHz and 5 kHz in the measured results.

Metamaterials are man-made structured nanomaterials composed of small structures that in bulk behave like a continuous material with unconventional effective properties. The acoustic analogue to electromagnetic metamaterials has already been validated in earlier works, and the possibility of extending the metamaterials concept to acoustic waves has already been investigated^1^. Building on this work, acoustic metamaterials have focused on developing artificial structures that can manipulate and control sound waves in unconventional ways^2^, made possible by the creation of unusual material properties such as double negativity (negative mass density and modulus)^3^,^4^, zero – or even negative – refractive index^5^,^6^,^7^,^8^, acoustic imaging^9^,^10^, cloaking^11^,^12^,^13^, transformation acoustics^14^, active acoustic metamaterials^15^, and acoustic metasurfaces^16^,^17^. As a fundamental research subject in acoustic metamaterials, element design has been focused primarily on two kinds of strategies – locally-resonant materials^3^,^18^ and effective medium theory based composites^19^. Recently, the design of geometry-based non-locally resonant elements has been theoretically proposed and experimentally verified^20^,^21^,^22^. These types of element can be designed to possess extreme parameters with low absorption and wide operating bandwidth. However, challenges still exist, including the development of efficient techniques for manufacturing metamaterial structures on a large scale and designing functional devices with excellent impedance matching.

In this paper, we design, fabricate, and demonstrate a new kind of acoustic metamaterial element with fractal geometry, which is inspired by the fractal features of geography in the natural world. Man-made examples involving fractal structures can be found in architectural designs and microwave devices^23^. Fractal acoustic metamaterial (FAM) is a tapered structure with self-similar mathematical description. Although they may appear complex, FAMs can be easily designed to obtain specific parameters through high resolution computer programs and can be reliably fabricated with existing rapid-prototyping technology. Extracted parameters from one-dimensional reflection-transmission measurements demonstrate good impedance match and broad operating bandwidth for the FAM element. To validate the performance of the FAM, we design, fabricate, and measure a two-dimensional focusing lens using FAM elements. The results of the experiment indicate excellent performance in terms of operating working bandwidth, refractive index, and effective impedance. Furthermore, the designed FAM offers application flexibility due to the light weight and low cost in manufacture, which adopts photopolymer resin or acrylonitrile butadiene styrene (ABS) rather than metal materials, such as brass, stainless steel, and silver. Our new strategy may offer an alternate route to the design of novel materials and devices in acoustic engineering in the future.

## Results

### Design of fractal acoustic metamaterials and retrieved effective parameters

**Figure 1:**
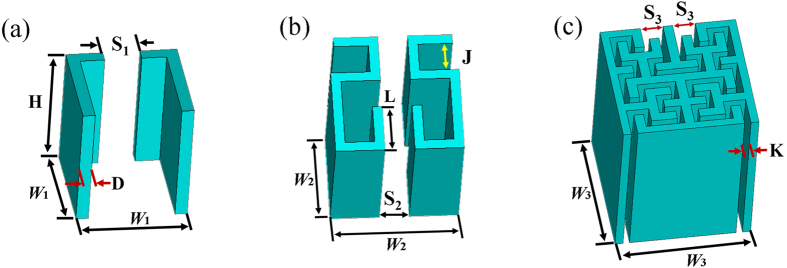
Illustration of the FAM element. Hilbert fractal elements for 0-, 1- and 2-order, where the height of all elements is *H* = 13 mm, and the thickness of photopolymer resin is *D* = 0.5 mm. (**a**) 1-order Hilbert FAM element, with *W*_*1*_ = 4.5 mm and *S*_*1*_ = 1.5 mm. (**b**) 2-order Hilbert FAM element, with *W*_*2*_ = 6.5 mm, *L* = 2.5 mm and *S*_*2*_ = 1.5 mm. (**c**) 3-order Hilbert FAM element, with *W*_*3*_ = 8.4 mm, *S*_*3*_ = 1.5 mm and *K* = 0.5 mm.

Two-dimensional spatial fractal structures, presented in Fig. 1a–c, illustrate Hilbert FAM elements of 1-order, 2-order, and 3-order, respectively. It is necessary to explain how varying degrees of parameters expected can be engineered through the FAM design. Up to now, coiling-up structures or folded channels have been employed for acoustic wave modulation. Different from previous works, where labyrinthine channels were used for folding space to realize extreme parameters, we take advantage of the classical Hilbert fractal structure’s strong space-folding capability to obtain the expected effective parameters. The structure is generated in an iterative fashion with the use of collinear transformations, which consists of a continuous line connecting the centers of a uniform background grid. Suppose the curve fills in a square section *S* on an external side, by increasing iteration levels *n*, the space between lines diminishes accordingly while the length of total perimeter *L*(n) increases, where *L*(n) can be written as *L*(*n*) = (2^*n*^ + 1)*S*. For example, if *n* = 2, the FAM structure is five-fold that of *n* = 0. The sound propagating path is rapidly iterated by increasing the iteration level of *n.* That means the curled structure forces the acoustic wave to travel in folded channels, enabling the path length of the wave to be multiplied. In order to realize broadband acoustic elements within desired spectrum, we add slits in the fractal elements to increase their resonant frequencies, and therefore the effective parameters (density and modulus) change slowly within the spectrum of interest as a result. In our design, we need to select the fractal order first according to the requirement of the refraction index (high refraction index usually requires high fractal order), and then the slit width should be adjusted carefully by optimization to avoid acoustic resonance in the operation bandwidth of the acoustic devices. Like all the coiling/folding acoustic meta-atoms^20^,^22^,^24^,^25^, such space-folding structures bring two advantages: first, they can create a high refractive index; second, they can generate a broadband response, whereas for traditional locally resonating structures, the desired parameters can only be found in a narrow frequency band around the resonating peak.

**Figure 2:**
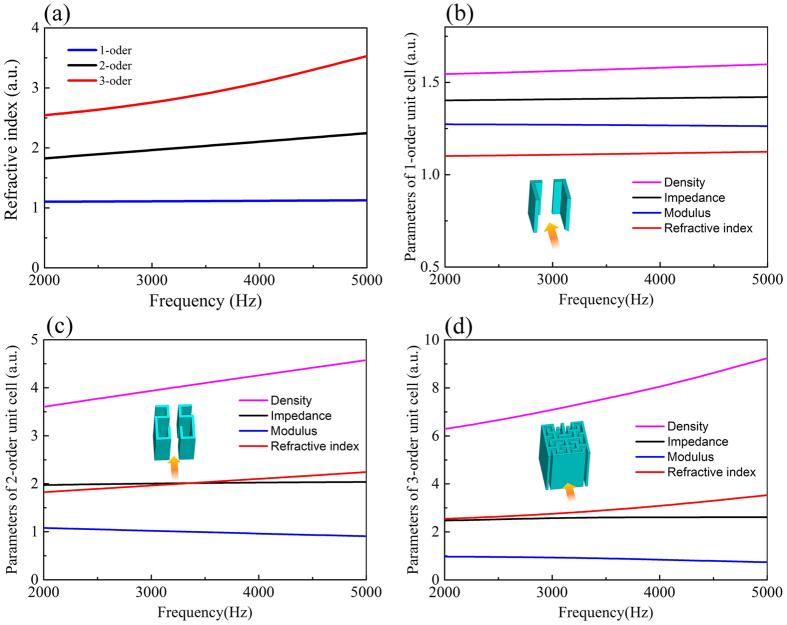
Effective parameters of FAM elements from 1-order to 3-order. (**a**) Effective refractive index. (**b**) Effective parameters of 1-order FAM element. (**c**) Effective parameters of 2-order FAM element. (**d**) Effective parameters of 3-order FAM element. The yellow arrows in Fig. 2a–d represent the propagation direction of the incident acoustic wave.

For the designed FAM element, the thickness (*D*) of materials in the Hilbert fractal structures is 0.5 mm. The height (*H*) of the cells is 13 mm, which is deposited on a 2 mm thick photosensitive resin plate. As shown in Fig. 1a, the slit space *S*_*1*_ is 1.5 mm in the 1-order FAM element with the periodicity *W*_*1*_ = 4.5 mm. For the 2-order FAM element shown in Fig. 1b, *W*_*2*_ = 6.5 mm, with *J* = 1.5 mm, *L* = 2.5 mm, *S*_*2*_ = 1.5 mm. The 3-order FAM element is shown in Fig. 1c, where *W*_*3*_ = 8.4 mm, *S*_*3*_ = 1.5 mm, and *K* = 0.5 mm. All the fabricated samples are made of photopolymer resin thermoplastic and fabricated via 3D printing (Stratasys Objet24, 0.05 mm precision). To demonstrate the performance of the FAM elements, the effective acoustic parameters are calculated utilizing a retrieval procedure based on commercial finite element analysis (FEA) solver COMSOL Multiphysics^26^,^27^. The effective density (*ρ*), bulk modulus (*B*), refractive index (*n*), and impedance (*z*) are illustrated in Fig. 2, showing weak dispersion from 2 kHz to 5 kHz. The effective refractive index *n* of elements with different orders changes slowly from 2 kHz to 5 kHz (Fig. 2b–d). In the meanwhile, the effective impedance *z* remains nearly constant for all three elements (Fig. 2b–d), which can be used to realize broadband impedance matching with various surrounding materials. The direction of the incident wave is indicated by the yellow arrow in Fig. 2. We here clarify that the effective parameters are only valid for one specific direction of incident wave and the background medium outside the Hilbert structures is assumed to be air in our design.

### Application: Acoustic focusing lens based on the FAM elements

As a potential application, we set out to design and fabricate an acoustic focusing lens by use of FAM elements. As discussed previously, by gradually increasing the fractal order, a gradient refractive index can be easily obtained as a result. The hyperbolic secant distribution, defined as *n*(*y*) = *n*_0_ sech(*αy*), has been proved to reduce the aberration of the focal spot, where *n*_*0*_ is the maximal refractive index at the center of the lens^28^,^29^. *α* denotes the gradient index coefficient with 
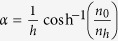
, where *n*_*h*_ refers to the minimal refractive index at the edge of the lens and *h* is half-height of the lens.

**Figure 3:**
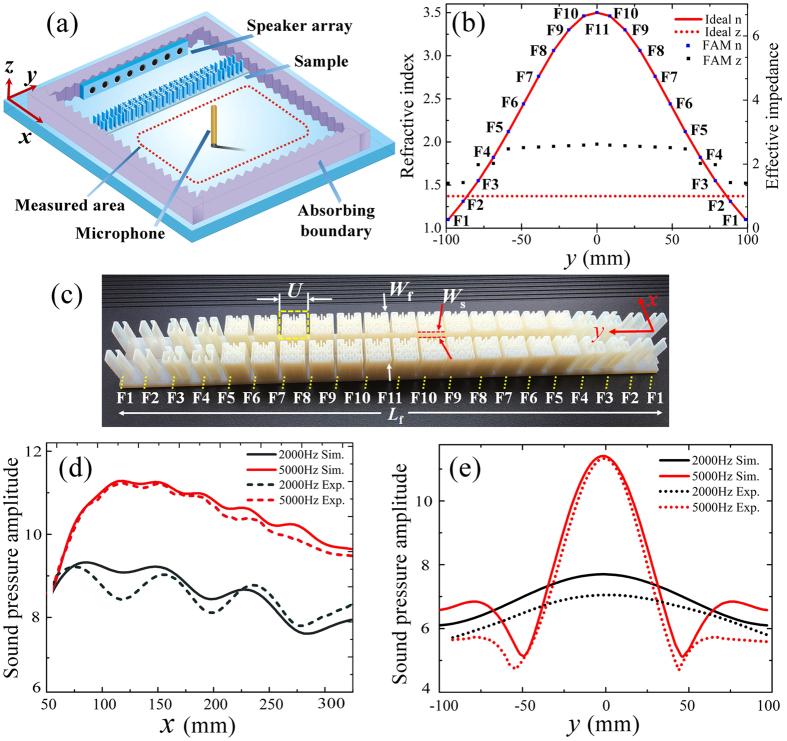
Experiment sample, simulated and measured results. (**a**) The schematic of the experimental setup. A plexiglass plate (not shown in the diagram) is located slightly above the sample. An eight-speaker array is used to generate a plane wave, and a microphone is used to sweep the area under test by a two-dimensional stepping motor and record the distributions of sound pressure around the sample. (**b**) The ideal refractive index and ideal impedance distributions required by the lens, and the available refractive index and realistic impedance distributions from the FAM elements. F1–11 represent FAM elements distributions required by the lens. (**c**) The lens prototype with *L*_*f*_ = 19.74 cm, *U* = 0.94 cm, *W*_*f*_ = 1.73 cm, and *W*_*s*_ = 0.5 mm. (**d**) The distribution of sound pressure amplitude along a line passing through the focus in the *x*-direction. (**e**) The distribution of sound pressure amplitude along a line passing through the focus in the *y*-direction.

**Figure 4:**
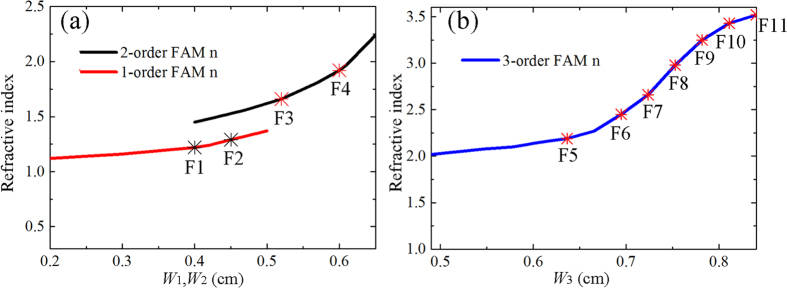
The relationship between the refractive index of FAM elements of different orders and the corresponding element width. The red, black (**a**) and blue (**b**) lines stand for the refraction index distributions of the 1^st^, 2^nd^, 3^rd^ -order FAM elements at 5 kHz by sweeping the element width *W*_*1*_*-W*_*3*_. The asterisks stand for the refraction index of the lens unit F1–F11 according to that required in Fig. 3(b).

From Fig. 2, the maximum and minimum refraction index of the FAM lens are 3.5 and 1.1 respectively. The lens can be discretized into 21 segments with the period 0.94 cm (Fig. 3c), covered by the elements F1-F11 with detailed material parameters and geometric dimensions listed in Table 1. To meet the requirement of the refraction index (Fig. 3b) for elements with different orders, the width *W*_*1*_, *W*_*2*_, and *W*_*3*_should be carefully adjusted in the design. For example, with the increase of *W*_*1,*_ the refraction index of the first order FAM elements grow slowly from 1 to 1.3 as shown in Fig. 4(a). By looking up Table 1, we can find that the elements F1 and F2 are actually 1^st^ FAM unit with corresponding *n* to be 1.21 and 1.37 respectively, whose corresponding element width *W*_*1*_ can be easily determined from the red curve in Fig. 4(a). As a result, the element width for other high order elements can also be achieved in a similar way, as indicated by asterisks in Fig. 4(a,b). The designed FAM focusing lens can be found in Fig. 3(c), where an ultrathin acoustic flat lens is obtained due to the high refractive index yielded by the fractal geometry structure. The thin focusing lens is composed by two layers, with the total thickness (*W*_*f*_) 1.73 cm, and the length of the lens (*L*_*f*_) 19.74 cm. All the orders of FAM elements have the same periodicity with *U* = 0.94 cm, which are aligned along the *y* direction.

To validate the performance of the FAM lens, both simulation and experiment have been carried out, as demonstrated in Fig. 3(d,e). During the numerical simulations, the perfect matched layers are used to prevent undesired reflections at the boundaries. The pressure field distributions at 2 kHz and 5 kHz along two orthogonal lines passing through the focus are extracted from the simulation, which is plotted in Fig. 3(d,e). The focal length is found to be 125 mm behind the lens and a narrow focusing spot can be clearly observed, which shows good focusing capability of the proposed FAM lens.

In the experiment, the two dimensional platform (detail information described in the methods section) is shown in Fig. 3(a), which is used to scan the sound pressure distributions within a plane slightly above the sample under test. The maxim scanning range is 120 mm × 180 mm, and the gap between the microphone and the sample is 0.5 mm. The measured data is also presented in Fig. 3(d,e) for comparison, showing excellent agreement with the simulation results. From the simulations data (see solid lines in Fig. 3(d,e)), we can calculate the sound pressure amplitude at the focal point relative to the reference sound pressure as 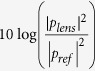 = 11.3 dB at 5 kHz and 9.2 dB at 2 kHz, which is easy to understand since the radiation aperture becomes larger with the increase of the operation frequency. From Fig. 3(d,e), good agreement between the simulation and experimental results is observed as expected, verifying the accuracy and reliability of FAM element design.

**Figure 5:**
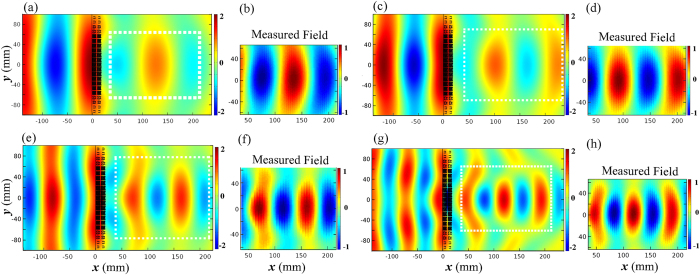
Distributions of the simulated and measured sound pressure around the FAM lens. (**a**) Simulated results at 2 kHz. (**b**) Measured results at 2 kHz. (**c**) Simulated results at 3 kHz. (**d**) Measured results at 3 kHz. (**e**) Simulated results at 4 kHz. (**f** ) Measured results at 4 kHz. (**g**) Simulated results at 5 kHz. (**h**) Measured results at 5 kHz.

To check the broadband property of the FAM focusing lens, the simulated and measured field distributions of the focusing lens are presented in Fig. 5 at 2 kHz, 3 kHz, 4 kHz, and 5 kHz respectively. From the simulated pressure distribution in Fig. 5 at different frequencies, the incident sound can be focused efficiently in a wide frequency range, and the focal spot is brighter and more concentrated when the frequency goes up, which is consistent to the analysis stated above. Due to limitation of our experimental platform, only a small region near the focus of the lens can be scanned, which is indicated by the white dashed lines in Fig. 5(a,c,e,g). The transmitted to incident power ratio is approximate to 5:1 at 5 kHz and 2:1 at 2 kHz from the experimental data. Comparing with the field-mapping results, the simulated results are in accordance with the actual conditions. However, there still exist small discrepancies at those frequencies that can be attributed to two reasons: one is sound leakage around the sample in the platform due to the unavailable small gaps between the sample and the upper plate, and the other is the accuracy of manufacture precision. To minimize the measurement error, a number of methods are employed in the experiment, including the use of elastic sound absorption materials to reduce the sound leakage, and high-precision microphone and transducer for detecting weak signals. The maximum impedance contrast between the lens and air appears at 5 kHz (see Fig. 5(g,h)), while the best impedance matching can be observed at 2 kHz (see Fig. 5(a,b)), since the large refractive index at the center of the lens usually requires larger dimensions of the FAM element at higher frequencies, which may deteriorate the impedance matching and in turn bring larger reflections at the lens surface.

## Conclusion

In summary, we propose and demonstrate a new kind of broadband FAM element for acoustic applications. By increasing the order of the fractal structure, it is easy to achieve high refractive index and therefore benefit the design of ultrathin acoustic devices such as lens and absorbers. This element shows non-resonant property with nearly constant bulk modulus and mass density at kilohertz frequencies. By carefully varying the dimensions of FAM element, a gradient refractive index can be achieved as a result. To demonstrate the performance of the resulting FAM elements, an acoustic focusing lens is proposed and experimentally verified. The lens is much thinner than previously reported results in the literature, due to the high refractive index achieved by the FAM element, which is especially useful for the design of novel acoustic devices in the future.

## Methods

### Numerical simulations

Numerical simulations based on finite element method (FEM) are carried out by COMSOL Multiphysics throughout this paper. In the numerical simulations, the frequency domain Helmholtz equation for sound pressure is solved in the air region, while in the photopolymer resin region the equation of structural mechanics for harmonic stresses and strains is solved. The acoustic waves are incident from the left side along *x* direction shown in Fig. 3(a), with the amplitude of sound pressure 1 Pa. The material parameters used in the numerical simulations are mass density of air *ρ*_0_ = 1.29 kg/m^3^, sound velocity in air *c*_0_ = 343 m/s, mass density of photopolymer resin *ρ*_s_ = 1300 kg/m^3^, sound velocity in photopolymer resin *c*_s_ = 716 m/s, bulk modulus of photopolymer resin *B*_*s*_ = 666.47 MPa, and Poisson’s ratio of photopolymer resin *σ* = 0.41.

### Field mapping measurement

The field mapping measurement is performed in a two-dimensional acoustic scanning platform. A linear 8-speaker array is used to generate a plane wave, and a microphone is used to scan the area under test and record the sound pressure distributions based on a 2D stepping motor controlled by a computer. The FAM elements are made of photopolymer resin thermal plastics and fabricated via 3D printing to meet the requirement of the model.

## Additional Information

**How to cite this article**: Song, G. Y. *et al*. Broadband Focusing Acoustic Lens Based on Fractal Metamaterials. *Sci. Rep.* **6**, 35929; doi: 10.1038/srep35929 (2016).

**Publisher’s note:** Springer Nature remains neutral with regard to jurisdictional claims in published maps and institutional affiliations.

## Figures and Tables

**Table 1:** Effective material parameters and structural dimensions of the lens elements.

FAM	F11	F10	F9	F8	F7	F6	F5	F4	F3	F2	F1
*Ideal n*	3.50	3.45	3.30	3.07	2.79	2.49	2.17	1.90	1.64	1.40	1.19
*FAM n*	3.50	3.43	3.25	3.03	2.74	2.45	2.19	1.92	1.66	1.37	1.21
*W/cm*	0.84	0.81	0.78	0.75	0.72	0.69	0.64	0.6	0.52	0.47	0.4
*U/cm*	0.94	0.94	0.94	0.94	0.94	0.94	0.94	0.94	0.94	0.94	0.94
*Order*	3rd	3rd	3rd	3rd	3rd	3rd	3rd	2nd	2nd	1st	1st
*FAM z*	2.62	2.59	2.57	2.55	2.53	2.51	2.47	2.03	1.98	1.42	1.4
